# Comparison between volunteer- and expert-led versions of a community-based weight-loss intervention

**DOI:** 10.1016/j.pmedr.2021.101370

**Published:** 2021-03-29

**Authors:** Ryoko Mizushima, Yoshio Nakata, Hiroyuki Sasai, Xinyu Zuo, Seiji Maeda, Kiyoji Tanaka

**Affiliations:** a Graduate School of Comprehensive Human Sciences, University of Tsukuba, Japan; b Department of Nutritional Epidemiology and Shokuiku, National Institute of Health and Nutrition, National Institutes of Biomedical Innovation, Health and Nutrition, Japan; c Faculty of Health and Sport Sciences, University of Tsukuba, Japan; d Research Team for Promoting Independence and Mental Health, Tokyo Metropolitan Institute of Gerontology, Japan; e THF Co., Ltd., Japan

**Keywords:** Body weight, Community-based, Weight-loss program, Obesity, BMI, body mass index, BOCF, baseline observation carried forward, CHW, community health worker, CI, confidence interval, DPP, Diabetes Prevention Program, FG, food group, HDL-C, high-density lipoprotein cholesterol, LDL-C, low-density lipoprotein cholesterol, MVPA, moderate to vigorous physical activity, TC, total cholesterol, UMIN, University Hospital Medical Information Network

## Abstract

This study compared the effect of volunteer- and expert-led versions of a community-based weight-loss intervention in a non-randomized comparative trial conducted in Ibaraki, Japan from 2016 to 2017. Participants were 145 Japanese adults with overweightness or obesity, aged 20–69 years, with 77 in a volunteer-led group and 68 in an expert-led group. Both groups received the same program content and intervention period. Community volunteers were trained in four or five 3-hour training sessions while experts were highly trained and experienced professionals in the fields of exercise and nutrition prescription. Participants were also instructed to maintain a well-balanced, low-energy diet. The primary outcome measure was body weight change. In the volunteer- and expert-led groups, 58 of 77 (75%) and 61 of 68 (95%) participants completed the 12-week intervention, respectively. The mean (95% confidence interval, CI) weight loss of the volunteer-led group was 6.4 (95% CI: 5.6–7.2) kg, corresponding to 8.9% of initial body weight, while that of the expert-led group was 6.3 (95% CI: 5.5–7.1) kg, corresponding to 8.2% of the initial body weight. The proportion of participants who completed the course was significantly higher in the expert-led group (*P* < 0.05); however, the degree of the body weight change was similar for both groups. With improvement in the completion proportion of the volunteer-led weight-loss interventions, such programs could be an alternative strategy for the wide-scale dissemination of low-cost obesity management.

## Highlights

• We compared the effects of volunteer- and expert-led weight-loss intervention.
• Participants were instructed to maintain a well-balanced, low-energy diet.
• The completion proportions was significantly higher in the expert-led group.
• The degree of body weight change was similar for both groups.
• Such programs could be an alternative strategy for low-cost obesity management.

## 1 Introduction

Obesity is currently a severe health problem worldwide (NCD, 1998). Large-scale clinical trials have demonstrated that lifestyle interventions emphasizing diet and physical activity decrease body weight (Ali et al., 2012, Aziz et al., 2015, Jenum et al., 2019, Knowler et al., 2002, Pedley et al., 2018). A typical program is derived from the Diabetes Prevention Program (DPP) (The Diabetes Prevention Program Research Group, 2002), which demonstrated that structured lifestyle interventions—such as training prediabetics to achieve modest weight loss through diet and physical activity—resulted in an average weight loss of 5.6 kg in 1 year (Knowler et al., 2002, The Diabetes Prevention Program (DPP) Research Group, 2002).

However, employing healthcare professionals to implement such lifestyle interventions is costly and may not be ideal, especially in communities with shortages of skilled healthcare workers and smaller budgets. In these cases, community health workers (CHWs) may be potential alternatives to healthcare professionals (Scott et al., 2018)—acting as a bridge between community residents and the local government, providing context-specific support, and producing improved long-term effects for local participants (Scott et al., 2018). For example, the DPP (The Diabetes Prevention Program Research Group, 2002) was transformed into a community-based intervention using CHWs (Hill et al., 2017, Katula et al., 2011, Katula et al., 2013, Koniak-Griffin et al., 2015, Norris et al., 2006, Ockene et al., 2012, Pedley et al., 2018, Sathish et al., 2013, Shah et al., 2013, Wilson et al., 2016, Yeary et al., 2020). Katula et al. (2011) implemented a CHW-led 12-month intervention for people with obesity and prediabetes and found significantly greater weight loss in the intervention group compared to the usual care control group.

A systematic review by Hill et al. (2017) reported the characteristics of CHWs involved in the DPP and their contributions to the expected outcomes. Of the 30 studies included in the analysis, 24 were conducted in the US, two each in India and New Zealand, and one each in Thailand and Australia. No studies from Japan were included. Studies were mainly conducted on minority populations and were set in predominantly community-based areas, such as churches, homes, and community centers. The CHWs were generally from the same local community as the residents and shared the same race or ethnicity and language backgrounds as the participants. The study designs included randomized controlled trials and quasi-experimental or comparative observational studies. The control groups received either usual care or no intervention. However, none of the studies directly compared CHW and expert-led interventions.

Ali et al. (2012) conducted a systematic review and *meta*-analysis of 28 US-based studies, which applied the DPP findings. The authors classified studies based on the type of personnel used to deliver the program: medical and allied health professionals, CHW-led, and electronic media-assisted methods. They found that each group of studies demonstrated significant weight loss at 12-month follow-up: 4.27 kg when led by medical and allied health professionals, 3.15 kg for CHW-led, and 4.20 kg for electronic media-assisted, with an overall mean loss of 3.99 kg. Accordingly, the authors concluded that CHW-led programs may have achieved similar weight loss as those led by medical and allied health professionals. However, these results were not based on entirely direct comparisons between CHW-led and expert-led interventions, but were merely literature comparisons of aggregated values; the comparability of interventions in each paper was not high. To address these gaps in existing literature, this study aimed to directly compare the outcomes between expert- and volunteer-led versions of a weight-loss intervention in Japan.

## 2 Measures

### 2.1 Design

This study was a non-randomized comparative trial comparing the change in body weight between participants in volunteer- and expert-led weight-loss intervention groups. To implement the volunteer-led weight-loss intervention, we recruited and trained community volunteers in Tsuchiura city (Ibaraki, Japan) as a part of a regional health service. This setting made it difficult to use a randomized controlled design. Therefore, we implemented the expert-led weight-loss intervention group in Tsukuba city (Ibaraki, Japan). The two cities are similar as they are adjacent to each other, located in the southern Ibaraki Prefecture, and are similar in population size (ranked 6th [Tsuchiura; population of 138,557] and 2nd [Tsukuba; population of 241,808] among 44 municipalities in Ibaraki Prefecture). The participants received no financial compensation. The University of Tsukuba ethical committee reviewed and approved the study protocol (No. 26-67 and 28-143), which was registered in the UMIN Clinical Trials Registry (UMIN000028214). This study is reported in accordance with the Transparent Reporting of Evaluations with Nonrandomized Designs (TREND) statement (Des Jarlais et al., 2004).

### 2.2 Community volunteers

Community volunteers in Tsuchiura city were recruited via local public relations magazines; recruitment flyers in city facilities, such as district public halls; and direct communication with existing volunteer organizations. Volunteers participated in a 1–2-month training course (4–5 sessions, 12–15 h in total); courses were held once a year for 3 years (2015–2017). After the training course, volunteers implemented a 12-week weight-loss intervention as many as three times from 2016 to 2017. In total, 41 volunteers coached a course at least once. The curriculum of the training course for community volunteers is described in Table 1 and included three basic didactic sessions and 1–2 applied and trial sessions. The same material sets, which included a textbook and notebook, developed for participants in the weight-loss intervention program were distributed to the volunteers. To foster a sense of group identity and cohesion among volunteers, a specially made polo shirt was also distributed.

**Table 1:** Curriculum of the training course for community volunteers (Ibaraki, Japan, 2016–2017).

Session 1 (didactic session)	
	Opening remarks and orientation
	Explanation of the weight-loss program
	Introduction of the textbook and notebook
Session 2 (didactic session)	
	Basics of weight-loss support: Part 1
	How to use the notebook
	Basics of the Four-Food-Group Point Method
	Application to the weight-loss program
Session 3 (didactic session)	
	Basics of weight-loss support: Part 2
	Tips about cooking to help weight loss
	Tips about weight-loss support
Session 4 (practice session)	
	Frame of mind as a community volunteer
	Practice reviewing the notebook
	Group work about writing feedback comments
Session 5 (practice session)	
	Rehearsal throughout a session
	Hearing about the actual experience of a weight-loss volunteer
	Group work about future activities
	Closing remarks

### 2.3 Participants

Participants for the volunteer- and expert-led weight-loss interventions were recruited through advertisements in a local newspaper. The eligibility criteria for the participants consisted of being between 20 and 69 years old and having a body mass index (BMI), calculated as weight in kilograms divided by squared height in meters, between 25 kg/m^2^ and 40 kg/m^2^. The exclusion criteria consisted of a history of coronary disease or stroke, or planned pregnancy. Participants provided written informed consent before baseline measurement.

The volunteer-led interventions were held three times in Tsuchiura city; the first group started in September 2016, while the second and third groups started in August 2017. In total, there were 77 study participants in the volunteer-led group. The expert-led interventions were held twice in Tsukuba city; both groups started in July 2017. The leader of the experts was one of the authors (R.M.), who is highly qualified (certified nutritionist and exercise instructor) and experienced in weight-loss support. The other 23 experts were post-doctoral research fellows and graduate students who had completed a training course for weight-loss management and had experience coaching adults with overweightness or obesity at least once. There were 112 applicants, of whom 68 met the inclusion criteria and constituted the expert-led group.

### 2.4 Weight-loss program

Participants in each group received identical weight-loss instructions from volunteers or experts. The educational materials in this study, such as the textbook and notebook, were based on prior work of the investigators (Tanaka et al., 2004, Nakata et al., 2011). The diet program was based on the Four-Food-Group Point Method (Kagawa, 1983). In brief, in the method, all foods are categorized into four food groups (FGs) based on their nutritional composition: FG1 (dairy products and eggs), FG2 (meat, fish, and beans), FG3 (vegetables and fruits), and FG4 (grains, oil, and sugar). To calculate energy intake and nutrient balance easily, every 80 kcal (335 kJ) of food is counted as one point in this method (Kagawa, 1983). Participants were instructed to eat a well-balanced, low-energy diet of approximately 1,680 kcal (7,029 kJ) per day for men and 1,200 kcal (5,021 kJ) per day for women. The distributed textbook described how to effectively eat a balanced diet. The participants kept a notebook in which they recorded every food they ate, body weight, daily steps walked, and their subjective health and mental conditions during the entire 12-week intervention period. The participants were encouraged to measure food weight, body weight, and number of daily steps using their own digital food scales, bathroom weighing scales, and pedometers that were not provided by the researchers. They were also instructed to calculate their daily energy intake and check their nutritional balance using the textbook and notebook.

### 2.5 Outcome measures

**Fig. 1:**
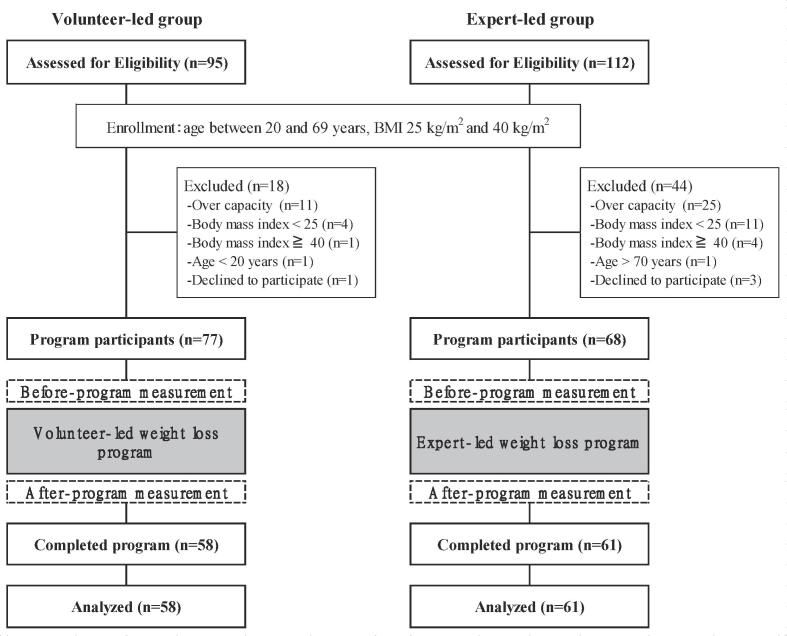
Participant flow chart from recruitment to end of program (Ibaraki, Japan, 2016–2017).

The primary outcome measure was the amount of weight loss from baseline to Week 12. The secondary outcome measures were changes in waist circumference, systolic blood pressure, diastolic blood pressure, triglycerides, high-density lipoprotein cholesterol (HDL-C), and fasting plasma glucose. These were measured in the morning after at least 12 h of fasting. Additionally, attendance and completion rate, socioeconomic factors, health characteristics, dietary intake, and physical activity were assessed. All data at baseline were collected after written informed consent was obtained and before the first intervention session started (Fig. 1).

#### 2.5.1 Weight change and attendance

Participants wore only their underwear and were barefoot during the anthropometric measurements. Body weight was measured to the nearest 0.1 kg using a digital scale (InBody 770; Biospace, Seoul, Korea), height was measured to the nearest 0.1 cm using a wall-mounted stadiometer, and BMI was calculated using these values. The percent of weight change was calculated by dividing the change in weight by the baseline weight and multiplying this by 100.

Attendance was calculated for those who participated in the program at least once as the percentage of sessions attended. A group mean was then calculated for each condition. The completion rate was calculated by dividing the participants who completed the post-intervention measurement by the total number of participants per group.

#### 2.5.2 Socioeconomic factors and baseline health characteristics

At baseline, socioeconomic factors, lifestyle habits, medical history, and medication use for hypertension, dyslipidemia, and diabetes were assessed using a questionnaire. Waist circumference at the level of the umbilicus in a standing position was measured directly on the skin to the nearest 0.1 cm using a measuring tape (in duplicate, then averaged). Body composition was estimated by a bioelectrical impedance device (HBF-306-W; Omron Healthcare, Kyoto, Japan), measured twice, and the average was used for the data analysis. Systolic and diastolic blood pressures were measured with an automated sphygmomanometer (Digital Automatic Blood Pressure Monitor HEM-762; Omron Healthcare). The measurements were taken on the right arm of seated participants who had rested for more than 5 min with the arm supported at heart level. The lower value of two readings was used for the data analysis.

#### 2.5.3 Blood biochemistry

A blood sample was drawn from each participant after an overnight fast (≥12 h), and the venous blood was assayed by an independent laboratory (Ibaraki Health Service Association and Tsukuba i-Laboratory LLP, Ibaraki, Japan). The measures of blood biochemistry were low-density lipoprotein cholesterol (LDL-C), HDL-C, triglycerides, total cholesterol (TC), fasting plasma glucose, hemoglobin, and hematocrit.

#### 2.5.4 Dietary intake and physical activity

Dietary intake was assessed by a food frequency questionnaire (Excel Eiyo-Kun FFQg ver. 4.0, Kenpakusha, Tokyo, Japan). Intake frequency in the last month of the intervention was reported on a weekly basis. The validity of this method has been verified (Sone et al., 2004). In this study, nutrient intake, nutrient-energy ratio, and intake of the four FGs (Kagawa, 1983) were calculated. Physical activity was assessed by Global Physical Activity Questionnaire, which has been validated and used worldwide as a standard physical activity questionnaire (Bull et al., 2009). The questionnaire measures time spent in moderate-to-vigorous physical activity (MVPA) within specific domains (work, travel, and recreation) and sedentary behavior.

### 2.6 Statistical analysis

Attendance was calculated for those who participated in the program at least once, and the remaining measurement items were analyzed for those who completed the pre- and post-intervention measurements. For the participants’ basic characteristics, continuous variables were reported as the mean (standard deviation), and categorical variables were reported as the number of participants (percentage). Within-group changes were reported as the mean (95% confidence interval [CI]). Analysis of covariance (ANCOVA) was used to examine the statistical significance of between-group differences, considering the respective baseline values. A Pearson’s chi-square test was then used to compare proportions. Additionally, only the primary outcome was analyzed in the dataset with the baseline observation carried forward (BOCF) for all participants enrolled in the program. Statistical analyses were performed using SPSS software, version 25.0 (IBM, Tokyo, Japan) with the level of statistical significance set at 5%.

## 3 Results

Fig. 1 shows the participant flowchart. In the volunteer-led group, all 77 participants attended the intervention sessions at least once; the mean attendance was 75% of sessions (range: 13%–100%). Within this group, 58 participants completed the 12-week intervention (75%), and their attendance was 88% of sessions (range: 38%–100%). In the expert-led group, all 68 members participated at least once, and the mean attendance was 81% of sessions (range: 38%–100%). Within this group, 61 participants completed the 12-week intervention (90%); their attendance was 85% of sessions (range: 38%–100%). The proportion of participants who completed the course was significantly higher in the expert-led group than in the volunteer-led group (*P* < 0.05).

The baseline characteristics of participants are shown in Table 2, Table 3, Table 4. In Table 2, a significant difference between groups was observed in height (*P* < 0.05), whereas BMI was similar for both groups. Other socioeconomic factors, lifestyle habits, and medical history did not differ between groups. In Table 3, systolic blood pressure, fasting plasma glucose, and hematocrit were significantly different between groups at baseline (*P* < 0.05), as was nutrient intake (*P* < 0.05; Table 4).

**Table 2:** Baseline characteristics of participants in the volunteer-led and expert-led groups (Ibaraki, Japan, 2016–2017).

	Volunteer-led group (n = 58)	Expert-led group (n = 61)	Group differences,
*P*-value			
Women, n (%)	44 (76)	38 (62)	0.11
Age, years	57.2 (10.3)	54.0 (11.2)	0.13
Height, cm	159.4 (8.4)	162.8 (9.3)	<0.05
Body weight, kg	72.5 (12.4)	76.7 (13.6)	0.08
BMI, kg/m^2^	28.4 (2.8)	28.8 (3.1)	0.51
BMI^1^ ≥ 25 kg/m^2^, n (%)	58 (100)	61 (100)	–
Sleep duration, hours/day	6.6 (1.3)	6.5 (0.9)	0.61
Current smoker, n (%)	4 (7)	5 (8)	0.79
Drinks alcohol, n (%)	34 (59)	33 (54)	0.62
Postmenopausal, n (%)	32 (55)	22 (36)	0.16
Employed full- or part-time, n (%)	31 (53)	40 (66)	0.18
College or vocational school graduate, n (%)	32 (55)	35 (57)	0.81
Household income greater than 5 million yen, n (%)	28 (48)	33 (54)	0.53
Lives with other people, n (%)	56 (97)	60 (98)	0.53
Medical history			
Hypertension, n (%)	21 (36)	14 (23)	0.11
Dyslipidemia, n (%)	13 (22)	8 (13)	0.18
Diabetes, n (%)	3 (5)	6 (10)	0.34
Medication use			
Antihypertensive, n (%)	21 (36)	14 (23)	0.11
Lipid-lowering, n (%)	3 (5)	1 (2)	0.29
Hypoglycemic, n (%)	1 (2)	4 (7)	0.19

Note: Data are expressed as mean (standard deviation) for baseline values.

1 BMI = body mass index.

**Table 3:** Changes in body weight, waist circumference, fat mass percentage, blood pressure, and blood biochemistry during the 12-week weight-loss program (Ibaraki, Japan, 2016–2017).

	Volunteer-led group (n = 58)		Expert-led group (n = 61)		Group differences, P-value	
	Baseline	Change	Baseline	Change	Baseline	Change
Body weight, kg	72.5 (12.4)	−6.4 (−7.2, −5.6)	76.7 (13.6)	−6.3 (−7.1, −5.5)	0.08	0.45
BMI^1^, kg/m^2^	28.4 (2.8)	−2.5 (−2.8, −2.2)	28.8 (3.1)	−2.3 (−2.6, −2.1)	0.51	0.24
BMI ≥ 25 kg/m^2^, n (%)	58 (100)	−24 (41)	61 (100)	−16 (26)	–	0.08
BMI ≥ 30 kg/m^2^, n (%)	17 (29)	−13 (22)	18 (30)	−9 (15)	0.98	0.28
Waist circumference, cm	97.5 (8.2)	−7.0 (−8.0, −6.0)	99.6 (8.3)	−8.3 (−9.2, −7.3)	0.18	0.11
Fat mass percentage, %	36.3 (4.3)	−2.4 (−3.0, −1.8)	34.6 (5.2)	−1.4 (−1.9, −0.9)	0.05	0.06
Blood pressure						
Systolic, mmHg	137.0 (13.0)	−7.4 (−11.3, −3.6)	130.6 (16.9)	−13.2 (−16.5, −9.8)	<0.05	<0.001
Diastolic, mmHg	88.2 (8.7)	−6.2 (−8.3, 4.1)	85.3 (11.2)	−6.9 (−8.7, −5.1)	0.10	0.18
Blood biochemistry						
LDL-C^2^, mmol/L	3.233 (0.804)	−0.144 (−0.339, 0.050)	3.480 (0.681)	−0.411 (−0.519, −0.303)	0.06	0.09
HDL-C^3^, mmol/L	1.479 (0.397)	0.086 (−0.021, 0.194)	1.438 (0.319)	−0.115 (−0.154, −0.075)	0.55	<0.001
Triglycerides, mmol/L	1.386 (0.728)	−0.358 (−0.574, −0.143)	1.365 (0.524)	−0.412 (−0.530, −0.294)	0.94	0.44
TC^4^, mmol/L	5.375 (0.897)	−0.173 (−0.398, 0.053)	5.498 (0.918)	−0.537 (−0.676, −0.397)	0.38	<0.01
Fasting plasma glucose, mmol/L	5.503 (0.837)	−0.217 (−0.436, 0.001)	5.788 (0.708)	−0.305 (−0.443, −0.166)	<0.05	0.31
Hemoglobin, hemoglobin	0.135 (0.012)	0.003 (0.000, 0.005)	0.138 (0.013)	−0.001 (−0.003, 0.001)	0.12	<0.05
Hematocrit, proportion	0.408 (0.035)	0.007 (−000.2, 0.016)	0.424 (0.034)	−0.004 (−0.009, 0.001)	<0.05	0.08

Note: Data are expressed as mean (standard deviation) for baseline values and as mean (95% confidence interval) for 12-week changes, unless otherwise specified.

1 BMI = body mass index.

2 LDL-C = low density lipoprotein cholesterol.

3 HDL-C = high density lipoprotein cholesterol.

4 TC = total cholesterol.

**Table 4:** Changes in nutrient intake and physical activity during the 12-week weight-loss program (Ibaraki, Japan, 2016–2017).

	Volunteer-led group (n = 58)		Expert-led group (n = 61)		Group differences, P-value	
	Baseline	Change	Baseline	Change	Baseline	Change
Nutrient intake						
Total energy intake, kJ/day	6690 (1671)	−1840 (−2290, −1390)	10,885 (3608)	−4244 (−5008, −3480)	<0.001	<0.01
Protein intake, g/day	69.8 (15.5)	−5.3 (−9.5, −1.1)	83.8 (25.3)	−20.7 (−26.8, −14.6)	<0.001	<0.05
% total energy intake	13.8 (2.2)	2.4 (1.9, 3.0)	13.0 (1.9)	3.0 (2.5, 3.4)	<0.05	0.71
Fat intake, g/day	68.7 (14.9)	−12.1 (−16.6, −7.6)	80.4 (26.0)	–22.5 (−29.0, −16.0)	<0.01	0.26
% total energy intake	30.6 (4.9)	1.1 (−0.2, 2.3)	28.0 (5.6)	4.4 (2.8, 6.0)	<0.01	<0.05
Carbohydrate intake, g/day	267.5 (68.4)	−67.9 (−84.1, −51.7)	362.4 (135.1)	−166.7 (−194.5, −138.9)	<0.001	<0.001
% total energy intake	55.6 (6.4)	−3.5 (−5.1, −1.9)	59.0 (7.1)	−7.4 (−9.3, −5.4)	<0.01	0.09
Four food groups (FGs)						
FG1 (dairy products and eggs), kJ/day	942 (389)	−81 (−174, 12)	1005 (536)	−140 (−242, −39)	0.46	0.61
FG2 (meat, fish, and beans), kJ/day	1673 (671)	–23 (−213, 168)	1967 (873)	−343 (−579, −107)	<0.05	0.30
FG3 (vegetable and fruits), kJ/day	634 (341)	131 (52, 210)	598 (341)	36 (−67, 140)	0.57	<0.05
FG4 (grains, oil, and sugar), kJ/day	5080 (1370)	−1835 (−2201, −1469)	7086 (3055)	−3749 (−4400, −3098)	<0.001	<0.05
Physical activity						
MVPA^1^, min/week	363 (531)	105 (−28, 239)	317 (450)	30 (−75, 136)	0.61	0.32
Work, min/week	134 (324)	30 (−65, 125)	85 (254)	34 (−49, 116)	0.36	0.72
Travel, min/week	105 (200)	34 (−16, 84)	125 (257)	−40 (−90, 10)	0.64	<0.05
Recreation, min/week	124 (197)	41 (−6, 88)	107 (158)	37 (−3, 77)	0.61	0.87
Sedentary, min/week	394 (216)	−60 (−104, −16)	438 (260)	11 (−41, 63)	0.31	<0.05

Note: Data are expressed as mean (standard deviation) for baseline values and as mean (95% confidence interval) for 12-week changes, unless otherwise specified.

1 MVPA = moderate to vigorous physical activity.

After the 12-week intervention, the average weight loss of the volunteer-led group was 6.4 (95% CI: 5.6–7.2) kg, corresponding to 8.9% of initial body weight (Table 3). In this group, 49 (84%) lost ≥ 5% and 28 (48%) lost ≥ 10% of their initial body weight. This was similar to the expert-led group, in which the average weight loss was 6.3 (95% CI: 5.5–7.1) kg, corresponding to 8.2% of initial body weight. In this group, 51 (84%) lost ≥ 5% and 15 (25%) lost ≥ 10% of their initial body weight. There was no significant difference in weight loss between groups (*P* = 0.45). Additionally, analysis of the BOCF datasets of all participants showed no significant difference between groups (*P* = 0.15); the average weight loss was 4.8 (95% CI: 3.9–5.7) kg in the volunteer-led group and 5.6 (95% CI: 4.8–6.5) kg in the expert-led group.

Significant within-group differences were observed in both groups regarding waist circumference, fat mass percentage, and systolic and diastolic blood pressures (Table 3). Although there were no significant between-group differences in the changes in waist circumference, fat mass percentage and diastolic blood pressure, systolic blood pressure decreased significantly more in the expert-led group than in the volunteer-led group.

Biochemical measures in the volunteer-led group did not change significantly except for triglycerides. In contrast, in the expert-led group, LDL-C, triglycerides, TC, and fasting plasma glucose significantly decreased, while HDL-C significantly decreased. Significant between-group differences were observed in changes in HDL-C, TC, and hemoglobin.

Regarding nutrient intake, total energy intake significantly decreased in both groups (Table 4). Change in total energy intake was significantly greater in the expert-led group. The carbohydrate-energy ratio was significantly reduced, and the protein-energy ratio was significantly increased in both groups. Comparing the 4 FGs, most changes were observed in FG4, which decreased significantly in both groups’ diets. However, noticeable changes in physical activity were not observed.

## 4 Discussion

The present study compared change in body weight between volunteer- and expert-led interventions, but only for participants who completed the 12-week program. There was no significant difference in weight loss between the groups, whereas the proportion of participants who completed the expert-led group intervention was significantly higher than that of the volunteer-led group.

A prior *meta*-analysis (Ali et al., 2012) showed that weight changes in participants receiving weight-loss interventions were similar regardless of whether interventions were delivered by experts or volunteers. However, this *meta*-analysis included some single-group pre- and post-studies; a comparison across these studies was difficult because differences between the participants in the different studies were not controlled. Further, the systematic review and *meta*-analysis of 30 studies by Hill et al. (2017) did not directly compare volunteer- and expert-led interventions. To address these issues, our study used a non-randomized controlled trial to directly compare weight loss in groups led by experts and by volunteers; to the best of our knowledge, this is the first study to do so. The results showed that weight loss was similar regardless of whether the group was led by experts or by volunteers, as found by Ali et al. (2012).

It is known that for diabetes prevention, weight reduction of 5% of initial body weight is necessary (The Diabetes Prevention Program (DPP) Research Group, 2002, Tuomilehto et al., 2001). The minimum weight loss required to improve obesity-related risk factors is reported to be 3% among Japanese persons (Muramoto et al., 2014). Most previous studies using volunteer-led interventions (Hill et al., 2017) achieved the 5–7% target weight loss of the DPP (The Diabetes Prevention Program Research Group, 2002). Our study, using a weight-loss program developed by Tanaka et al. (2004), achieved 8.9% weight loss, which is similar and even slightly higher than that reported in previous studies (Hill et al., 2017). Thus, this weight loss was sufficient and clinically significant for a volunteer-led weight loss intervention program.

However, the proportion of participants who completed the course of the volunteer-led weight-loss group in this study was 75%, which was significantly lower than the 90% in the expert-led group (*P* < 0.05). In previous studies, completion proportions were reported to be 93% (Hill et al., 2017), 88% (Koniak-Griffin et al., 2015), or 76% (Ackermann et al., 2008). From these results, the completion proportion in the current study was similar or somewhat inferior to the previous studies. Therefore, future efforts should be directed at increasing participants’ completion proportions. The reason why completion was lower for the volunteer-led group is probably due to the experience value of the instructors. As a solution, the training course period for community volunteers should be extended. In previous studies, the duration of the training varied considerably among studies—from a single one-hour session to even 100 h or more (Hill et al., 2017, Wilson et al., 2016, Koniak-Griffin et al., 2015, Sathish et al., 2013). It should be noted that studies with short training course periods were mainly worksite-based or pilot studies (Hill et al., 2017, Wilson et al., 2016). In the present study, the training course ranged from 12 to 15 h in total, which has scope for improvement.

Most secondary outcomes in this study were similarly improved in both groups, although some items showed significant differences. The degree of improvement was similar to our previous studies (Tanaka et al., 2004, Nakata et al., 2011) and those of others (Hill et al., 2017). No significant changes were found in the amount of physical activity of the participants in both groups; however, significant improvements were seen in the amount of energy intake. Therefore, the observed weight loss was mainly derived from changes in diet. The carbohydrate-energy ratio was significantly reduced, and the protein-energy ratio was significantly increased in both groups. Of the four FGs, the most change was observed in FG4. The dietary program used in the study recommended reducing FG4 intake and eating only necessary quantities of FGs 1–3. The observed changes in nutrient intake followed the dietary instructions and suggested high compliance with the dietary program in both groups.

A previous study that examined the cost-effectiveness of a CHW-led weight-loss program showed that its cost was approximately one-third that of the expert-led intervention, whereas the effect on body weight was similar (Lawlor et al., 2013). The present study did not actually verify cost-effectiveness; however, the cost of implementing the volunteer-led group of this study is likely to be much lower than that of employing health professionals. Therefore, implementation in similar community settings has the potential to produce important public health benefits.

### 4.1 Strengths and limitations

For studies conducted outside clinical settings, it can be challenging to incorporate appropriate control groups in the study design. This study’s strength was that it used a controlled comparison between expert- and volunteer-led versions of the same community-based weight-loss intervention.

However, there were some limitations. First, the study design was not a randomized controlled trial since the participants in the two groups were recruited separately from different cities. At baseline, no significant differences were found in most measured variables between the groups, such as sex, age, body weight, and BMI. However, some baseline characteristics were significantly different between groups, such as height, blood pressure, blood biochemistry, and total energy intake. These differences suggest the presence of potential selection bias in the study. These items can affect changes in dietary intake and body weight. A future perspective is necessary to design randomized controlled trials to verify equivalence or non-inferiority. Second, the 12-week intervention period was relatively short with no follow-up measures. Maintaining weight after an initial weight-loss period is clinically important (Knowler et al., 2009, Tuomilehto et al., 2001). Most people with overweightness or obesity regain their weight over time (Wing et al., 2016). Therefore, future studies should compare the long-term effectiveness of the volunteer- vs. expert-led interventions. Third, we measured the participants’ dietary intake using a food frequency questionnaire. However, this may underestimate total energy intake along with carbohydrate, fat, and protein intakes (Naska et al., 2017). Fourth, the validity of the measurements was not ideal. We used a simple measurement method to ensure feasibility; thus, the accuracy of some outcomes may have been inferior (e.g., fat mass percentage, nutrient intake, and sedentary behavior). Finally, this study was conducted in a specific area of Japan. Therefore, to increase findings’ generalizability, similar studies in different locations are required.

## 5 Conclusions

We implemented a volunteer-led, community-based weight-loss intervention and compared body weight changes between volunteer- and expert-led interventions. Similar weight loss was observed in both groups, and no significant difference was found. However, the proportion of participants who completed the volunteer-led intervention was lower than that of the expert-led intervention. With an improvement in completion, volunteer-led weight-loss interventions could become an alternative strategy for the wide-spread dissemination of a low-cost obesity management program.

## Author contributions

Ryoko Mizushima, Yoshio Nakata, and Hiroyuki Sasai were responsible for planning the study design, acquiring funds, managing intervention research, training volunteers, leading expert interventions, analyzing data, interpreting the data, and writing the first draft. Xinyu Zuo played an important role in data collection, including in the management of intervention study, training of volunteers, and guidance in expert interventions. Seiji Maeda and Kiyoji Tanaka oversaw all aspects of the study. All authors reviewed the article and contributed to its final form.

## Declaration of Competing Interest

The authors declare the following financial interests/personal relationships which may be considered as potential competing interests: Kiyoji Tanaka is a founder and president of a university-originated startup company named THF Co., Ltd. The authors outsourced part of the volunteer training programs to this company and purchased the textbook and notebook they published for volunteer training and group-based weight loss programs.

## References

[b0005] Ackermann R.T., Finch E.A., Brizendine E., Zhou H., Marrero D.G. (2008). Translating the diabetes prevention program into the community. The DEPLOY pilot study. Am. J. Prev. Med..

[b0010] Ali M.K., Echouffo-Tcheugui J., Williamson D.F. (2012). How effective were lifestyle interventions in real-world settings that were modeled on the Diabetes Prevention Program?. Health Aff. (Millwood)..

[b0015] Aziz Z., Absetz P., Oldroyd J., Pronk N.P., Oldenburg B. (2015). A systematic review of real-world diabetes prevention programs: learnings from the last 15 years. Implement. Sci..

[b0020] Bull F.C., Maslin T.S., Armstrong T. (2009). Global physical activity questionnaire (GPAQ): nine country reliability and validity study. J. Phys. Act. Health.

[b0025] Des Jarlais D.C., Lyles C., Crepaz N. (2004). Improving the reporting quality of nonrandomized evaluations of behavioral and public health interventions: the TREND statement. Am. J. Public Health.

[b0030] Hill J., Peer N., Oldenburg B., Kengne A.P., Krukowski R.A. (2017). Roles, responsibilities and characteristics of lay community health workers involved in diabetes prevention programmes: a systematic review. PLoS One.

[b0035] Jenum A.K., Brekke I., Mdala I., Muilwijk M., Ramachandran A., Kjøllesdal M., Andersen E., Richardsen K.R., Douglas A., Cezard G., Sheikh A., Celis-Morales C.A., Gill J.M.R., Sattar N., Bhopal R.S., Beune E., Stronks K., Vandvik P.O., van Valkengoed I.G.M. (2019). Effects of dietary and physical activity interventions on the risk of type 2 diabetes in South Asians: meta-analysis of individual participant data from randomised controlled trials. Diabetologia.

[b0040] Kagawa A. (1983). The ‘four-food-group-point-method’ (in Japanese). J. Kagawa Nutr. Univ..

[b0045] Katula J.A., Vitolins M.Z., Morgan T.M., Lawlor M.S., Blackwell C.S., Isom S.P., Pedley C.F., Goff D.C. (2013). The healthy living partnerships to prevent diabetes study: 2-year outcomes of a randomized controlled trial. Am. J. Prev. Med..

[b0050] Katula J.A., Vitolins M.Z., Rosenberger E.L., Blackwell C.S., Morgan T.M., Lawlor M.S., Goff D.C. (2011). One-year results of a community based translation of the Diabetes Prevention Program: Healthy-Living Partnerships to Prevent Diabetes (HELP PD) Project. Diab. Care.

[b0055] Knowler W.C., Barrett-Connor E., Fowler S.E. (2002). Reduction in the incidence of type 2 diabetes with lifestyle intervention or metformin. N. Engl. J. Med..

[b0060] Knowler W.C., Fowler S.E., Hamman R.F. (2009). 10-year follow-up of diabetes incidence and weight loss in the Diabetes Prevention Program Outcomes Study. Lancet.

[b0065] Koniak-Griffin D., Brecht M.L., Takayanagi S., Villegas J., Melendrez M., Balcázar H. (2015). A community health worker-led lifestyle behavior intervention for Latina (Hispanic) women: feasibility and outcomes of a randomized controlled trial. Int. J. Nurs. Stud..

[b0070] Lawlor M.S., Blackwell C.S., Isom S.P., Katula J.A., Vitolins M.Z., Morgan T.M., Goff D.C. (2013). Cost of a group translation of the Diabetes Prevention Program: healthy living partnerships to prevent diabetes. Am. J. Prev. Med..

[b0075] Muramoto A., Matsushita M., Kato A., Yamamoto N., Koike G., Nakamura M., Numata T., Tamakoshi A., Tsushita K. (2014). Three percent weight reduction is the minimum requirement to improve health hazards in obese and overweight people in Japan. Obes. Res. Clin. Pract..

[b0080] Nakata Y., Okada M., Hashimoto K., Harada Y., Sone H., Tanaka K. (2011). Comparison of education-only versus group-based intervention in promoting weight loss: a randomised controlled trial. Obes. Facts.

[b0085] Naska A., Lagiou A., Lagiou P. (2017). Dietary assessment methods in epidemiological research: current state of the art and future prospects. F1000Res.

[b0090] NCD Risk Factor Collaboration (NCD-RisC) (1998). Trends in adult body-mass index in 200 countries from 1975 to 2014: a pooled analysis of 1698 population-based measurement studies with 19·2 million participants. Lancet.

[b0095] Norris S.L., Chowdhury F.M., Van Le K., Horsley T., Brownstein J.N., Zhang X., Jack L., Satterfield D.W. (2006). Effectiveness of community health workers in the care of persons with diabetes. Diab. Med..

[b0100] Ockene Ira S., Tellez Trinidad L., Rosal Milagros C., Reed George W., Mordes John, Merriam Philip A., Olendzki Barbara C., Handelman Garry, Nicolosi Robert, Ma Yunsheng (2012). Outcomes of a Latino community-based intervention for the prevention of diabetes: the Lawrence Latino Diabetes Prevention Project. Am. J. Public Health.

[b0105] Pedley C.F., Case L.D., Blackwell C.S., Katula J.A., Vitolins M.Z. (2018). The 24-month metabolic benefits of the healthy living partnerships to prevent diabetes: a community-based translational study. Diab. Metab. Syndr..

[b0110] Sathish Thirunavukkarasu, Williams Emily D, Pasricha Naanki, Absetz Pilvikki, Lorgelly Paula, Wolfe Rory, Mathews Elezebeth, Aziz Zahra, Thankappan Kavumpurathu Raman, Zimmet Paul, Fisher Edwin, Tapp Robyn, Hollingsworth Bruce, Mahal Ajay, Shaw Jonathan, Jolley Damien, Daivadanam Meena, Oldenburg Brian (2013). Cluster randomised controlled trial of a peer-led lifestyle intervention program: study protocol for the Kerala diabetes prevention program. BMC Public Health.

[b0115] Scott Kerry, Beckham S.W., Gross Margaret, Pariyo George, Rao Krishna D, Cometto Giorgio, Perry Henry B. (2018). What do we know about community-based health worker programs? A systematic review of existing reviews on community health workers. Hum. Resour. Health.

[b0120] Shah M., Kaselitz E., Heisler M. (2013). The role of community health workers in diabetes: update on current literature. Curr. Diab. Rep..

[b0125] Sone H., Yoshimura Y., Ito H., Ohashi Y., Yamada N. (2004). Japan Diabetes Complications Study Group. Energy intake and obesity in Japanese patients with type 2 diabetes. Lancet.

[b0130] Tanaka K., Okura T., Shigematsu R. (2004). Target value of intraabdominal fat area for improving coronary heart disease risk factors. Obes. Res..

[b0135] The Diabetes Prevention Program (DPP) Research Group (2002). The Diabetes Prevention Program (DPP) Description of lifestyle intervention. Diab. Care.

[b0140] Tuomilehto Jaakko, Lindström Jaana, Eriksson Johan G., Valle Timo T., Hämäläinen Helena, Ilanne-Parikka Pirjo, Keinänen-Kiukaanniemi Sirkka, Laakso Mauri, Louheranta Anne, Rastas Merja, Salminen Virpi, Aunola Sirkka, Cepaitis Zygimantas, Moltchanov Vladislav, Hakumäki Martti, Mannelin Marjo, Martikkala Vesa, Sundvall Jouko, Uusitupa Matti (2001). Prevention of type 2 diabetes mellitus by changes in lifestyle among subjects with impaired glucose tolerance. N. Engl. J. Med..

[b0145] Wilson M.G., DeJoy D.M., Vandenberg R.J., Corso P., Padilla H., Zuercher H. (2016). Effect of intensity and program delivery on the translation of diabetes prevention program to worksites: a randomized controlled trial of fuel your life. J. Occup. Environ. Med..

[b0150] Wing R.R., Espeland M.A., Clark J.M. (2016). Association of weight loss maintenance and weight regain on 4-year changes in CVD risk factors: the action for health in diabetes (Look AHEAD) clinical trial. Diab. Care.

[b0155] Yeary Karen H. Kim, Kaplan Cameron M., Hutchins Ellen (2020). Implementation costs of a community health worker delivered weight loss intervention in black churches serving underserved communities. Prev. Med. Rep..

